# MicroRNAs in the pathogenesis of neurodegenerative disorders: Potential as therapeutic targets

**DOI:** 10.4103/NRR.NRR-D-25-00402

**Published:** 2025-07-05

**Authors:** Aditi Singh, Manivannan Subramanian, Amit Singh

**Affiliations:** 1 Department of Biology, University of Dayton, Dayton, OH, USA; 2 Premedical Program, University of Dayton, Dayton, OH, USA; 3 Leonard A. Mann Endowed Chair in Natural Sciences, University of Dayton, Dayton, OH, USA; 4 Center for Genomic Advocacy (TCGA), Indiana State University, Terre Haute, IN, USA

**Keywords:** Alzheimer’s disease, amyotrophic lateral sclerosis, *Drosophila*, gene expression regulation, genetics, Huntington’s disease, microRNA, neurodegenerative diseases, Parkinson’s disease

## Abstract

Neurodegenerative diseases (neurodegenerative disorders) are marked by the progressive degeneration of the structure and function of the central nervous system. They may result in the deterioration of cognitive, motor, and functional abilities. Diseases such as Alzheimer’s disease, Parkinson’s disease, Huntington’s disease, and amyotrophic lateral sclerosis represent some of the most prominent examples of neurodegenerative disorders. Despite scientific advancement in understanding disease pathology and prognosis, the therapeutic strategies available for management remain limited. In recent years, microRNAs, small non-coding RNA molecules, have emerged as key players in the pathogenesis of neurodegenerative disorders. Therefore, understanding how these microRNAs affect disease pathology and pathway signaling is essential, and may open microRNAs as new avenues for potential therapeutic intervention. This review explores the role of microRNAs in various neurodegenerative diseases, discuss how microRNAs affect signaling pathways, and examine the potential of microRNAs as therapeutic targets.

## Introduction

Despite only 3% of the human genome encodes for proteins, the remaining 76% transcribed gives rise to a significant portion of noncoding RNAs (ncRNAs) including microRNAs (miRNAs). These ncRNAs can play important roles in development, differentiation, and disease progression (Ali et al., 2024). ncRNAs are an umbrella term that defines several noncoding RNAs that are linked to functional roles in modulating cellular processes. ncRNAs include miRNAs, small nucleolar RNAs, and PIWI-interacting RNAs (Cai et al, 2009). Unlike proteins, these ncRNAs do not require a long post-translational process to drive their function. Therefore, they can vary in size, number, and capacity to cause change(s). As such, understanding how ncRNAs play a role in cell function in normal and abnormal pathophysiology is an area of scientific investigation. This review aims to further analyze how a subset of ncRNAs, miRNAs, function in the background of neurodegenerative disorders and their potential as therapeutic targets.

**Figure 1:**
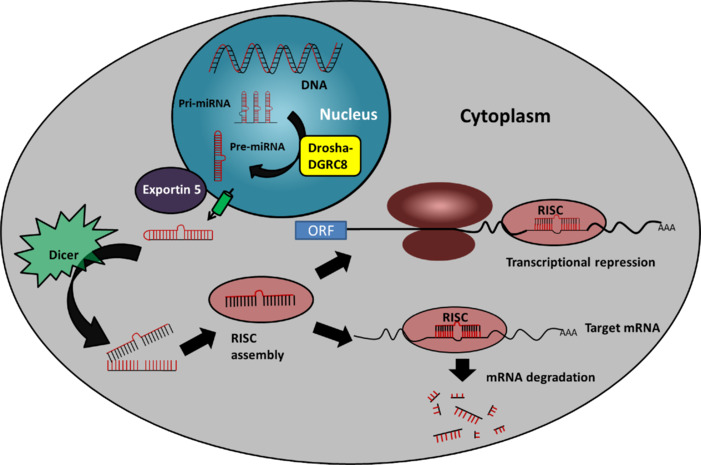
Gene regulation by miRNAs. Primary miRNA will be synthesized in the nucleus which undergoes cleavage to pre-miRNA by the RNase called Drosha-DGRC8. The pre-miRNA will be transported to cytosol and undergo further processing to mature miRNAs. These mature miRNAs have seed sequences that are complementary to their target genes. The mature miRNAs will bind to 3’UTR sequences of their target mRNAs and regulate the expression of their target genes either by transcriptional repression or by degrading the mRNAs. DGCR8: DiGeorge syndrome critical region gene 8; RISC: RNA-induced silencing complex; UTR: un-translated region.

miRNAs are short (approximately 22 nucleotides long), highly conserved single-stranded, ncRNA molecules that regulate gene expression primarily at the post-transcriptional level. Primary miRNA is synthesized in the nucleus and is cleaved into pre-miRNA by the RNase called Drosha-DGRC8 (**Figure 1**). This pre-miRNA is then transported to the cytosol and continues to undergo further post-transcriptional processing to form the mature miRNAs (**Figure 1**). These mature miRNAs exert their regulatory effect by binding to complementary sequences within the 3’ untranslated region (3’UTR) of target messenger RNAs (mRNAs). This binding can lead to either degradation of the targeted mRNA or inhibition of the targeted mRNA’s translation (**Figure 1**). miRNAs have been implicated in regulating a wide range of biological processes such as development, differentiation, apoptosis, and stress responses (Ambros, 2004; Bartel, 2004; Aranha et al., 2011; Giusti et al., 2014; Feng et al., 2017).

In neurons, miRNAs are essential to maintaining synaptic plasticity, neurogenesis, neuronal differentiation, and neuronal survival (Ballas et al., 2005; Dajas-Bailador et al., 2012; De Gregorio et al., 2018). miRNA dysfunction, often caused by genetic mutations, epigenetic alterations, or cellular stress, can impair cellular processes and contribute to the onset and progression of neurodegenerative disorders (Cai et al., 2009). Understanding how miRNAs associate with neurodegenerative disorders and how multiple miRNAs may interact to promote disease is a frontier in unraveling the science behind neurodegenerative disorders. The ability of miRNAs to regulate multiple genes within complex pathways makes them particularly attractive candidates for understanding disease mechanisms and developing novel therapeutic strategies. This review will define the role of miRNAs in Neurodegenerative disorders, describe how they alter signaling pathways, and discuss the therapeutic potential of targeting miRNAs in neurodegenerative disorders.

## Search Strategy

All literature searches were carried out on the PubMed database using these keywords: Alzheimer’s disease, amyotrophic lateral sclerosis, Drosophila, gene expression regulation, genetics, Huntington’s disease, microRNA, neurodegenerative diseases, Parkinson’s disease. All years were included in the search.

## Role of MicroRNAs in Neurodegenerative Disorders

MiRNAs are thought to play a modulatory role in neuronal differentiation, cell fate specification, neuronal proliferation, and homeostasis (Cai et al., 2009; Juźwik et al., 2019; Li et al., 2023). When dysregulated, miRNAs can promote neurodegenerative disorders via genetic mutations, epigenetic modifications, oxidative stress induction or cellular dysfunction, and neuroinflammation (Ballas et al., 2005; Jauhari et al., 2018; Deshpande et al., 2024). Mutations in genes encoding miRNAs or in their target mRNAs can lead to altered miRNA expression patterns. Such mutations can affect overall neuronal survival and synaptic transmission, which are important for neuronal homeostasis. Additionally, epigenetic modifications such as DNA methylation and histone modifications can also alter miRNA expression. Pathological processes such as oxidative stress, protein misfolding, or mitochondrial dysfunction can also disrupt miRNA expression and allow for neuronal vulnerability. Such chronic neuroinflammation exposes neurons to pathological insults and can modulate miRNA levels. Thus, aberrant miRNA signaling can result in unintended post-translational modifications in precursors to some proteins associated with neurodegenerative disorders. Given that miRNAs regulate a wide umbrella of mechanisms to maintain neuronal homeostasis, understanding how these molecules respond in times of distress, such as neurodegenerative disorders, can shed light on how miRNAs impact disease.

### Alzheimer’s disease

Alzheimer’s disease (AD) is characterized by the accumulation of neuroinflammatory plaques due to extracellular deposition of amyloid-beta 42 protein (Aβ_42_) and/or the intracellular deposition of neurofibrillary tangles, composed of hyperphosphorylated tau proteins in hippocampal synaptic populations in the brain (Absalon et al., 2013; Cutler et al., 2015; Sarkar et al., 2016; Deshpande et al., 2019). Both Aβ_42_ plaques and hyperphosphorylated tau tangles contribute to the disruption of synaptic transmission and present as neuronal death at the cellular level and cognitive decline symptomatically (Banzhaf‐Strathmann et al., 2014; Singh et al., 2024). Various miRNAs have been implicated in regulating the molecular pathways underlying AD, especially those involved in amyloid precursor protein (APP) processing, tau phosphorylation, neuroinflammation, and synaptic function.

Studies using cell lines, fruit flies, mouse models, and postmortem AD brains have revealed interactions between miRNAs and numerous genes implicated in AD pathogenesis (Liu et al., 2012; Sarkar et al., 2016; Konovalova et al., 2019; Yeates et al., 2019; Barros-Viegas et al., 2020). Notably, miRNAs bind to the 3′UTR of key target genes involved in AD development, such as *APP*, Presenilin 1 (*PSEN1*), Presenilin 2 (*PSEN2*), and Apolipoprotein E (*APOE*) (Konovalova et al., 2019). miRNAs regulate APP and Aβ synthesis through various mechanisms. For instance, miR-29 targets *BACE1*, an enzyme that promotes Aβ formation from *APP* (Barros-Viegas et al., 2020). The downregulation of miR-29 in AD patients contributes to increased Aβ levels. Similarly, another miRNA downregulated in AD is miR-106b, which represses the lipid transporter *ABCA1*, thereby reducing Aβ formation (Liu et al., 2016). Alternatively, neurotypical levels of miR-106b suppress *APP* synthesis that prevents excessive Aβ accumulation. Given that miRNAs play a modulatory role in several inflammatory and functional proteins, understanding which miRNA(s) promote disease pathology is a novel area of interest. This review has identified a few key miRNAs that influence the genetic expression and post-transcriptional changes associated with AD pathology.

#### miR-9

**Figure 2:**
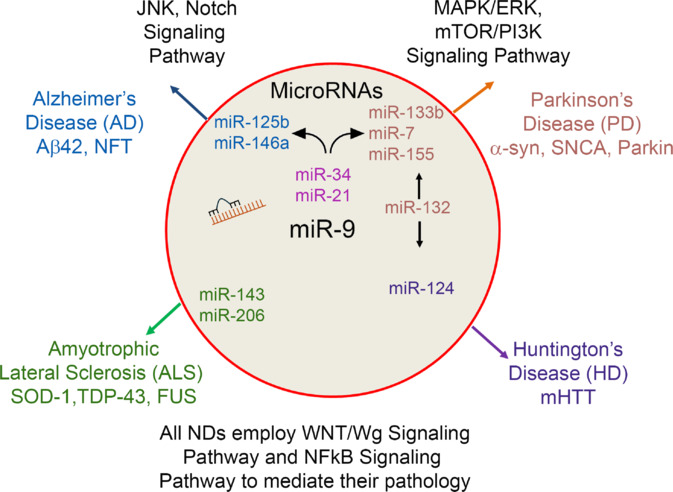
MiRNAs as modulators in neurodegenerative disease and signaling pathways. MiRNAs have been identified as modulators for several different Neurodegenerative disorders. A compilation of the miRNAs that are associated with various Neurodegenerative disorders and signaling pathways. miR-9 is involved in modulating aspects of AD, PD, ALS, and HD. miR-34 and miR-21 contribute to post-translational regulation of both AD and PD. Similarly, miR-132 is involved in similar modifications for PD and HD. Other miRNAs such as the cohort of miR-125b and miR-146a are associated with AD; miR-133b, miR-7, and miR-155 mediate PD pathology; miR-124 aids in HD pathogenesis; and ALS is mediated by miR-143 and miR-206. Each of these miRNAs can also modulate signaling pathway readouts corresponding to associated Neurodegenerative disorders and ND-mediated pathology. Created with BioRender.com. Aβ: Amyloid-beta; ERK: extracellular signal-regulated kinase; FUS: Fused in Sarcoma; JNK: c-Jun N-terminal kinase; MAPK: mitogen-activated protein kinase; mHTT: mutant huntingtin protein; miRNAs: microRNAs; mTOR: mammalian target of rapamycin; ND: neurodegeneration; NFT: neurofibrillary tangle; NF-κB: nuclear factor-κB; PI3K: phosphatidylinositol 3-kinase; SNCA: synuclein alpha; SOD-1: superoxide dismutase 1; TDP-43: TAR DNA-binding protein 43; α-syn: α-synuclein.

miR-9 is one of the most abundantly expressed miRNAs in the developing larval and adult brain (Cai et al., 2009; Coolen et al., 2013; Alwin Prem Anand et al., 2020; **Figure 2** and **Table 1**). Not only is miR-9 highly conserved evolutionarily, but miR-9 also plays a fundamental role in stabilizing the proliferation and differentiation of embryonic neural stem cells and neural progenitor cell (NSC/NPC) populations (Coolen et al., 2013). In AD, miR-9 has been shown to regulate APP processing, modulating the production of Aβ peptides. Lower expression of miR-9 has been observed in AD brains, contributing to increased APP levels and Aβ accumulation (Chen et al., 2021). Elevated expression of miR-9 is associated with enhanced progenitor proliferation and differentiation. The role of miR-9 in determining cell fate via modulating proliferation and differentiation occurs through its large targeting capacity. The targets of miR-9 include Notch signaling effectors, *Hes*, and a cohort of transcription factors, including forkhead transcription factor FoxG1, the homeobox factor Gsx2, the orphan nuclear receptor Tlx/Nr2e, and the zinc finger transcription factor Zic5 (Coolen et al., 2013; Alwin Prem Anand et al., 2020). The involvement of mature miR-9-5p in regulating neuronal fate in neurodegenerative disorders is further evidenced by its interaction with the transcription factor RE1-Silencing Transcription Factor (REST) (Giusti et al., 2014), which suppresses neuronal differentiation while promoting miR-9-5p expression (Ballas et al., 2005; Hwang and Zukin, 2018). In turn, miR-9-5p targets and inhibits REST, forming a negative feedback loop that modulates neural fate. Given the vast role of miR-9, this miRNA plays a role in modulating several targets associated with different neurodegenerative disorders.

**Table 1:** MicroRNAs, diseases, and signaling pathways: A summary

Diseases	MicroRNAs	Signaling pathways	References
Alzheimer’s disease	•miR-9 •miR-34a •miR-125b •miR-146a •miR-21t	•Wnt/Wg •MAPK/ERK •NF-κB •Notch	Aranha et al., 2011; Coolen et al., 2013; Banzhaf‐Strathmann et al., 2014; Saba et al., 2014; Jauhari et al., 2018
Parkinson’s disease	•miR-133b •miR-7 •miR-34b/c •miR-132 •miR-21 •miR-155	•Wnt/Wg •MAPK/ERK •NF-κB •mTOR/PI3K	Choi et al., 2014; Kim et al., 2014; De Gregorio et al., 2018; Konovalova et al., 2019; Bai and Bian, 2022
Huntington’s disease	•miR-124 •miR-132 •miR-9	•Wnt/Wg •NF-κB	Packer et al., 2008; Johnson and Buckley, 2009; Konovalova et al., 2019; Smith-Geater et al., 2020
Amyotrophic lateral sclerosis	•miR-9 •miR-143 •miR-206	•Wnt/Wg •NF-κB	Di Pietro et al., 2017; Waller et al., 2017; Hawley et al., 2019; Ammal Kaidery et al., 2021; Jiang et al., 2021

This table summarizes the microRNAs involved in modulating Alzheimer’s disease, Parkinson’s disease, Huntington’s disease, and amyotrophic lateral sclerosis. It also outlines the signaling pathways (e.g., Wnt/Wg, MAPK/ERK, and NF-κB) regulated by microRNAs. The table includes references for studies that have discussed these individuals or cohorts of microRNAs and corresponding signaling pathways. ERK: Extracellular signal-regulated kinase; MAPK: mitogen-activated protein kinase; mTOR: mammalian target of rapamycin; NF-κB: nuclear factor-κB; PI3K: phosphatidylinositol 3-kinase.

#### miR-34a

The miR-34 family is highly expressed throughout the adult mammalian brain throughout neurogenesis. miR-34a is involved in regulating NSC/NPC differentiation, neuronal maturation, and maintaining mature neuron population (Agostini et al., 2011; Aranha et al., 2011; Choi et al., 2011; De Antonellis et al., 2011; Jauhari et al., 2018). During brain development, miR-34 plays a dual role in either maintaining cells in a proliferative state or promoting differentiation, depending on which genes miR-34 targets (Aranha et al., 2011; Choi et al., 2011). This delicate balance is crucial for proper NSC/NPC function and brain development. miR-34 has a broad range of biological effects and many targets in mature neurons. Therefore, miR-34 expression is a tightly regulated process. In AD, elevated miR-34a levels lead to tau hyperphosphorylation through targeting the *SIRT1* gene, a deacetylase that regulates tau stability (Aranha et al., 2011). This targeting results in tau aggregation and neuronal dysfunction. When miR-34 is inhibited in AD mouse models, improvements in cognitive impairment via enhancing synaptic plasticity and reducing amyloidogenic processing of APP are observed (Aranha et al., 2011).

#### miR-125b

miR-125b can modulate the expression of genes involved in synaptic function and neuroinflammation (Banzhaf‐Strathmann et al., 2014; Liu et al., 2024). Synaptically, miR-125b targets the NR2A subunit of NMDA receptors and weakens channel efficacy (Banzhaf‐Strathmann et al., 2014). As a result, neurons cannot open voltage or neurotransmitter-based channels long enough for potentiation, and neuronal conductance is affected. miR-125b is also considered a pro-inflammatory regulator, although the mechanism remains understudied. Preliminary evidence suggests that miR-125b potentiates inflammatory signaling pathways (Liu et al., 2024). In AD, miR-125b promotes tau hyperphosphorylation by activating p35, cdk5, and p44/42 mitogen-activated protein kinase (MAPK) signaling while downregulating the dual specificity phosphatase 6 and protein phosphatase 1 catalytic subunit alpha in both *in vitro* and *in vivo* models (Banzhaf‐Strathmann et al., 2014). Overexpression of miR-125b also inhibits neuronal proliferation while exacerbating apoptosis, inflammation, and oxidative stress in *in vitro* AD models (Zhuang et al., 2020; **Figure 2** and **Table 1**).

#### miR-146a

miR-146a likely prevents an overstimulated inflammatory response through regulating components of the nuclear factor-κB (NF-κB) pathway (Saba et al., 2014; **Figure 2** and **Table 1**). miR-146a modulates Toll-like receptors, which consequently mediate the downstream activation of inflammatory pathways such as NF-κB, MAPK, and members of the interferon regulatory factor family that all produce proinflammatory cytokines. While NF-κB activates pre-miR-146a, the mature miR-146a-5p inhibits IRAK1 and tumor necrosis factor receptor-associated factor 6, two critical NF-κB signaling components that act as a brake on excessive immune activation (Saba et al., 2014). When miR-146 expression is reduced, loss of this regulation results in severe motor dysfunction, a ten-fold increase in myeloid cell proliferation, and heightened T-cell activation (Saba et al., 2014). In AD, this miRNA contributes to chronic neuroinflammation and generates a highly redox-sensitive environment by targeting genes that are involved in the NF-κB pathway (Lei et al., 2021). Oxidatively sensitive environments are prone to trigger aberrant activation of signaling pathways that result in neuronal cell death (Deshpande et al., 2024). Interestingly, mature miR-146a-5p may play a neuroprotective role in neurodegenerative disorders since its overexpression promotes axon remyelination via changes in macrophage polarization (Sun et al., 2023).

#### miR-277

**Figure 3:**
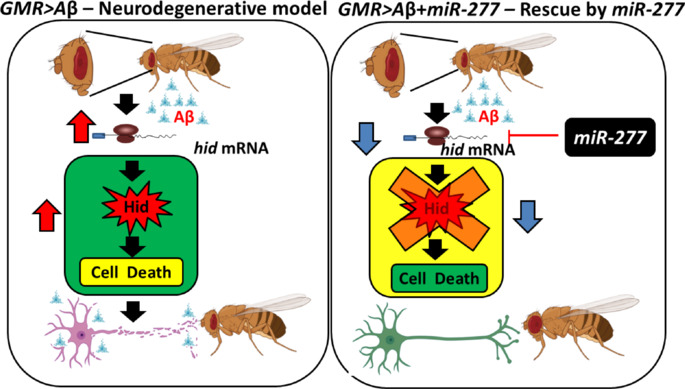
GOF of *miR-277* in the *Drosophila* neurodegenerative model system ameliorates the neurodegenerative phenotype of GMR > Aβ _42_. Misexpression of Aβ_42_ results in the accumulation of amyloid plaques with aberrant activation of the Hid signaling cascade, which eventually leads to cell death and neurodegeneration. GOF of *miR-277* in the *Drosophila* neurodegenerative model (GMR > Aβ_42_) results in the rescue of neurodegeneration by binding to 3’UTR of *hid* mRNA and reduces its expression and cell death. 3’UTR: 3’ Untranslated region; Aβ: amyloid-beta; GMR: glass multiple repeat; GOF: gain-of-function; Hid: head involution defective.

MiRNAs are essential for gene regulation and play a crucial role in various biological processes, leading to diseases such as AD. In a forward genetic screen using the *Drosophila* eye model, mir-277 was identified as a genetic modifier that rescues Aβ_42_-mediated neurodegeneration in *Drosophila* (Deshpande et al., 2024; **Figure 3**). It was found that miR-277 is expressed in key tissues and serves as a neuroprotective agent that rescues neurodegeneration both at morphological as well as functional levels. Furthermore, miR-277 gain-of-function restores axonal targeting defects and prevents cell death as seen in developing *Drosophila* eye and the pupal retina. miR-277 targets the head involution defective (hid) proapoptotic gene that is regulated by the evolutionarily conserved Jun-N-terminal kinase (JNK) pathway known to be activated in AD (Tare et al., 2011; Gogia et al., 2020, 2021). Since this information has been generated in the genetically versatile model of *Drosophila*, which shares similar genetic machinery to humans, the potential of this miR-277 as a biomarker and therapeutic target of AD is promising. miR-277 has a human ortholog hsa-miR-3660, which is implicated in several human diseases, however, its role in AD has not yet been explored. There is a strong possibility that hsa-miR-3660 targets components of other apoptotic pathways. Therefore, more studies on miR-277 and its human ortholog are warranted to find insights into the mechanism of AD and other neurodegenerative disorders.

### Parkinson’s disease

Parkinson’s disease (PD) is characterized by the progressive loss of dopaminergic neurons in the substantia nigra located in the midbrain (Jankovic, 2008; Juźwik et al., 2019; Khezri et al., 2022; Li et al., 2023; Narwal et al., 2023). These dopaminergic neurons have projections that travel into the striatum and down to the basal ganglia (Jankovic, 2008). This connection is responsible for relaying information from the cerebral cortex to the cerebellum for movement planning and execution. Therefore, when these dopaminergic neurons display dysfunction or degradation, individuals with PD present with tremors, rigidity, and difficulty with movement (Jankovic, 2008). PD causality has a sporadic arm and hereditary arm that consists of mutations associated with the *SNCA*, *Parkin*, and *PINK1* genes (Zhang et al., 2017; Juźwik et al., 2019). As PD progresses, dopaminergic neurons degrade, and the formation of Lewy bodies, which contain aggregates of α-synuclein, marks the onset of PD-associated dementia (Emre, 2003). miRNAs influence several key processes associated with the progression and pathogenesis of PD including mitochondrial function, oxidative stress, neuroinflammation, and protein degradation pathways.

#### miR-133b

miR-133b is one of the most studied miRNAs in PD (**Figure 2** and **Table 1**). Evidence showing how miR-133b directly modulates PD-associated genes highlights various mechanism(s) at play. However, how these individual puzzle pieces fit together at the onset of PD remains unclear. miR-133b has been found to suppress midbrain dopaminergic neuron (DN) differentiation in the substantia nigra via targeting pituitary homeobox 3, a transcription factor (Kim et al., 2007; Zhang et al., 2017). Overexpression of miR-133b displayed a reduction in DAT transcription, a plasma membrane carrier in DN that functions as a late maturation marker in the midbrain (Kim et al., 2007). However, early midbrain transcription factor markers such as pituitary homeobox 3 and nuclear receptor-related factor 1 were unchanged (Kim et al., 2007). Logically, miR-133b knockouts show normal numbers of midbrain DN throughout development (Zhang et al., 2017). In PD, these DNs are compromised, and decreases in serum miR-133b levels are a consistent feature (Zhang et al., 2017). Overexpression of miR-133b reduces mature DN number; ergo, the fidelity of communication between the striatum and basal ganglia is compromised.

#### miR-7

miR-7 is thought to play a neuroprotective role by reducing α-synuclein-mediated apoptosis (Junn et al., 2009; Choi et al., 2014; Fragkouli and Doxakis, 2014; **Figure 2** and **Table 1**). Specifically, miR-7 inhibits proapoptotic proteins Bax and Sirt2 (Junn et al., 2009). If miR-7’s inhibition of these two proteins is simultaneous or if Sirt2 inhibition also promotes Bax inhibition remains unclear. However, both are mechanisms that allow for neuroprotection of DN. miR-7 also targets RelA, a component NF-κB pathway, and impairs the ability of RelA to affect downstream effector molecules that lead to cell death (Choi et al., 2014). In PD, the impaired activity of the DN mitochondrial electron transport chain’s complex 1 plays a role in propagating neuronal stress that culminates in neuronal cell death (Choi et al., 2014). Here, miR-7 levels are reduced and therefore cannot exert their neuroprotective activity. With reduced miR-7 levels, α-synuclein levels are elevated and form toxic aggregates that disrupt neuronal function (Fragkouli and Doxakis, 2014). Typically, miR-7 would inhibit neuronal death by upregulating mTOR pathway targets; however, miR-7 levels are endogenously low in PD and cannot exert their actions (Junn et al., 2009). The mechanism explaining low endogenous miR-7 levels in PD remains unclear and is an area of continued study.

#### miR-34b/c

miR-34b and miR-34c are two miRNAs that are associated with maintaining the integrity of the mitochondrial membrane and regulating levels of oxidative stress (Liu et al., 2012; Kabaria et al., 2015; Jauhari et al., 2018; **Figure 2** and **Table 1**). They are often clustered together as they functionally act similarly in a neuronal background. Interestingly, miR-34b/c have independent binding sites that directly target the α-synuclein’s 3’UTR and reduce the levels of intact protein expressed (Kabaria et al., 2015). When miR-34b/c levels are reduced, reduced mitochondrial function and increased oxidative stress are observed in neurodegenerative disorders (Konovalova et al., 2019). Decreased miR-34b/c are correlated with decreased Parkin and Parkinsonism-associated deglycase (*DJ-1*) expression (Konovalova et al., 2019). Changes in these protein expression profiles are associated with reduced mitochondrial function and allow for reactive oxygen species accumulation (Konovalova et al., 2019). Further, as members of the miR-34 family, they share some redundancy with miR-34a and miR-499a/b/c, and act on targets of the *Wnt* signaling pathway (De Gregorio et al., 2018). In particular, miR-34b/c is known to downregulate *Wnt1* and influence midbrain DN differentiation (De Gregorio et al., 2018). In PD, both miRNAs are downregulated in the substantia nigra, amygdala, and cerebellum, which suggests miR-34b/c target genes involved in PD pathogenesis. miR-34b has two binding sites while miR-34c has a single binding site (Kabaria et al., 2015). Therefore, overexpression of miR-34b/c reduces α-synuclein expression while conversely, miR-34b/c mutants display increased α-synuclein with the formation of α-synuclein aggregates.

#### miR-132

miR-132 and its variants, including miR-132-3p, have been associated with regulating neuroinflammation and associated DN degeneration (Hirsch and Hunot, 2009; Gong et al., 2022; **Figure 2** and **Table 1**). miR-132 has been shown to activate microglia as a part of its proinflammatory effect (Zhang et al., 2017; Gong et al., 2022). miR-132 represses the Glutaredoxin (GLXR) protein associated with managing oxidoreduction reactions that is highlighted in the context of neurodegenerative disorders (Gong et al., 2022). By doing so, miR-132 repressed GLXR cannot suppress microglial cell activation and allows for a massive inflammatory response in the form of apoptosis, neuronal degeneration, and potential onset of neurodegenerative disorders. In PD, the neuronal environment progresses into an inflamed and highly oxidative state where miR-132 can be credited for promoting this environment. Further, miR-132 microglial activation releases pro-inflammatory factors such as interleukin-6, interleukin-1b, and tumor necrosis factor-α that exacerbate neuronal injury (Qazi et al., 2021; Gong et al., 2022). Studies show that overexpression of miR-132 drives microglial-induced inflammation, leading to increased apoptosis, worsening of motor deficits, and progression of PD. Knockdown of miR-132 reduces cytokine release and promotes oxidative re-stabilization (Gong et al., 2022). Further, overexpression of miR-132 also inhibits SIRT1, a histone deacetylase of the sirtuin family (Qazi et al., 2021). In doing so, SIRT1 expression decreases and the population of acetylated p53 increases. Acetylated p53 induces apoptosis.

### Huntington’s disease

Huntington’s disease (HD) is an autosomal dominant neurodegenerative disorder (ND) characterized by a progressive decline in motor and cognitive function. Individuals present with uncontrolled jerky movements called chorea. HD is caused by a trinucleotide repeat (CAG) expansion in the *HTT* gene on chromosome 4 that leads to the production of a mutant huntingtin protein with an expanded polyglutamine tract (Johnson et al., 2008; Johnson and Buckley, 2009; Morigaki and Goto, 2017; Konovalova et al., 2019). This mutant protein aggregates and interferes with cellular processes, including gene transcription, protein degradation, and cellular signaling (Johnson et al., 2008; Lee et al., 2017; Morigaki and Goto, 2017; Fukuoka et al., 2018). Mutant huntingtin also leads to the atrophy of the caudate nucleus and in turn, signaling coming to and from the striatum into the basal ganglia is affected (Chang et al., 2017; Morigaki and Goto, 2017). miRNAs involved in regulating synaptic plasticity, neuroinflammation, and neuronal survival are a novel area of scientific exploration in HD.

#### miR-124

During development, miR-124 is upregulated to promote neurogenesis (Lee et al., 2017; Han et al., 2020; **Figure 2** and **Table 1**). miR-124 has also been found to fine-tune microglial quiescence (Han et al., 2020). Further, miR-124 levels remain high in post-mitotic neurons, suggesting a function in synaptic plasticity and memory signaling maintenance (Han et al., 2020). Reduced expression of miR-124 is observed in neurodegenerative disorders can lead to disruption in neuronal functions. For instance, miR-124 targets the methyl CpG binding protein 2 (*MeCP2*) gene to maintain synaptic plasticity (Johnson and Buckley, 2009; Konovalova et al., 2019; Han et al., 2020). MeCP2 will repress the nuclear transcription of proteins that coax neurons into maturing and allow for synapses to remain heavily branched (Packer et al., 2008). In HD, miR-124 is reportedly downregulated, and this results in increased levels of its target genes including *HTT* and proteins such as REST (Lee et al., 2017; Han et al., 2020). REST expression is the highest NSC and decreases when these cells develop into NPC (Packer et al., 2008). Mutant huntingtin does not silence REST activity; therefores REST can travel to the nucleus and bind to the RE1/neuron restrictive silencer element and cause transcriptional dysfunction (Packer et al., 2008; Johnson and Buckley, 2009; Lee et al., 2017). Meanwhile, overexpression of miR-124 decreases the REST protein expression and improves neuronal outcomes (Lee et al., 2017).

#### miR-9

As previously discussed, miR-9 is an abundantly expressed miRNA that plays a critical role in neuronal differentiation and development (Cai et al., 2009; Coolen et al., 2013; **Figure 2** and **Table 1**). In HD, miR-9 is downregulated, which results in increased mislocalization of the REST transcription factor. The miR-9 directly targets the 3’UTR of the REST mRNA and its cofactor, REST corepressor 1’s (CoREST) (Packer et al., 2008). Once REST arrives in the nucleus, REST can further dysregulate neuronal gene expression and lineage commitment through its direct control of mRNA transcription by recruiting corepressors such as CoREST and MeCP2 (Packer et al., 2008).

### Amyotrophic lateral sclerosis

Amyotrophic lateral sclerosis (ALS) is characterized by the progressive degeneration of upper and lower motor neurons associated with voluntary muscle contraction (Gogia et al., 2020; Han et al., 2020; Cheng et al., 2023; **Figure 2** and **Table 1**). ALS is manifested as muscle weakness, paralysis, muscle wasting, dementia, and eventual respiratory failure. Depending on the location of the initial motor loss, ALS can be characterized as either limb-onset, which is associated with loss of voluntary muscle contraction in specific limb(s), or bulbar onset that is associated with difficulty speaking or swallowing (Cheng et al., 2023). Furthermore, it is known that the origin of ALS can be classified under two arms: sporadic ALS (sALS) and familial ALS (fALS) (Konovalova et al., 2019; Gogia et al., 2020; Liu et al., 2022; Cheng et al., 2023). In sALS, the cause of the disease is thought to be rooted environmentally and is difficult to identify. Alternatively, fALS possesses a hereditary component where fALS is often associated with mutations in genes such as Fused in Sarcoma (*FUS*) and RNA-binding proteins such as transactive response DNA-binding protein 43 (TDP-43) that mislocalize and aggregate in the cytoplasm of neurons (Anderson et al., 2018; Cheng et al., 2023). miRNAs are known to be involved in motor neuron survival; therefore, are a good tool to study to understand as a potential therapeutic marker.

#### miR-143

miR-143 regulates the expression of genes involved in muscle maintenance. miR-143 matures into miR-143-3p and when upregulated likely suppresses myoblast differentiation and maturation (Du et al., 2016; Waller et al., 2017; **Figure 2** and **Table 1**). This finding suggests that miR-143-3p may inhibit myotube differentiation and maturation. Conversely, when miR-143 is downregulated, it promotes myoblast differentiation and contributes to muscle-wasting diseases (Waller et al., 2017). Such downregulation is observed in neurodegenerative disorders such as ALS where miR-143 levels are affected, which promotes muscle atrophy and loss of function. In ALS, miR-143 was observed in the serum of sALS patient samples and studies postulate that miR-143 may be correlated with increased muscle denervation of motor neurons during disease progression (Waller et al., 2017; Joilin et al., 2019). miR-143-3p binds to TDP-43, an RNA binding protein involved in neurodegenerative disorders such as ALS, affects the production of mature miR-143 expression and its downstream targets (Freischmidt et al., 2013; Anderson et al., 2018). This TDP-43 association shows how miR-143 also plays a role in modulating fALS.

#### miR-206

miR-206 is a skeletal muscle-specific miRNA and a repressor for brain-derived neurotrophic factor (Liu et al., 2023; **Figure 2** and **Table 1**). Brain-derived neurotrophic factor can cross the blood-brain barrier and affect components of the central nervous system. Therefore, miR-206 upregulation, as seen in ALS patients, can result in brain-derived neurotrophic factor downregulation, which causes neurodegeneration and deficits in neurogenesis (Liu et al., 2023). miR-206 suppresses histone deacetylase 4 (HDAC4). HDAC4 plays a role in increasing skeletal muscle during denervation and simultaneously blocking the reinnervation signaling (Di Pietro et al., 2017). In ALS, miR-206 suppression results in HDAC4 downregulation, which hastens motor neuron dysregulation. Alternatively, miR-206 overexpression boosts neuromuscular synapse regeneration after an acute nerve injury (Williams et al., 2009; Di Pietro et al., 2017; Liu et al., 2023). In a study examining miR-206 expression during various disease stages, it was shown that miR-206 expression levels are higher during the early stages of ALS and lower during later stages (Di Pietro et al., 2017). These findings suggest that miR-206 has specific targets that are associated with onset and early ALS progression, but fewer targets associated with later pathology and disease maintenance.

#### miR-9

Again, miR-9 plays a role in gene targeting upon ND pathology and is profusely expressed in the brain during early neuronal development. As previously discussed, miR-9 also functions in neuronal differentiation, fate, and development (Cai et al., 2009; Coolen et al., 2013; **Figure 2** and **Table 1**). In ALS, miR-9 is downregulated in motor neurons of the central nervous system in the spinal cord and targets 3’UTR of neurofilament proteins NFL, NFM, and NFH, alongside the peripherin protein. These proteins are integral for the formation of neuronal cytoplasmic inclusions or stress granules (Coolen et al., 2013; Hawley et al., 2019). Interestingly, miR-9 levels are upregulated in fALS and target the TDP-43 mRNA. Specifically, miR-9 is dysregulated in stressed neurons from patients carrying the *TARDBP A90V* mutation, likely due to reduced levels of TDP-43 (Hawley et al., 2019). Consistent with this, shRNA-mediated knockdown of TDP-43 in mouse primary cortical neurons also led to a decrease in miR-9 expression. Together, miR-9 expression varies by the type of ALS and the location of its protein targets. Therefore, while miR-9 is a good marker for ALS-based neurodegeneration, it is nonspecific.

## MicroRNAs and Signaling Pathways

miRNAs are critical for regulating cellular processes such as differentiation, survival, apoptosis, and stress response (Cai et al., 2009; Coolen et al., 2013; Juźwik et al., 2019; Li et al., 2023; **Figure 2** and **Table 1**). Signaling pathways are often involved in propagating or modulating these same cellular processes (Tare et al., 2011; Kim et al., 2014; Wittkorn et al., 2015; Gogia et al., 2020; Irwin et al., 2020; Yeates et al., 2023). In neurons, miRNAs regulate several signaling pathways that are involved in synaptic function, neuroinflammation, protein homeostasis, and mitochondrial function. Aberrant signaling in these pathways is commonly associated with neurodegenerative disorders. Dysregulated miRNA expression can exacerbate changes in signaling and contribute to ND onset, pathophysiology, and/or progression (Deshpande et al., 2024). The interplay between miRNAs and key signaling pathways in neurodegenerative disorders has become a subject of intense research. A novel approach in this field is to understand if miRNAs are not only modulating mature mRNAs, but also modulating other miRNAs (Packer et al., 2008). Understanding how miRNAs impact larger protein cascades such as signaling pathways can help us show how they most effectively promote neurodegenerative disorders. The overarching goal of the field is to elucidate the molecular mechanisms underlying neurodegeneration, with a particular focus on determining the role of miRNAs in these neurodegenerative disorders. Furthermore, neurodegenerative disorders are not singular where a single defect results in disease. Several defects cause a snowball of change by varying how innate signaling pathways are expressed. These changes can manifest in the forms of misfolded proteins, degeneration of specific proteins, and changes in synaptic transmission and neuronal signaling (Sheinerman et al., 2017; Konovalova et al., 2019; Li et al., 2023). This upcoming section will explore the role of miRNAs in known signaling pathways that have been identified as key pathways for ND pathology and progression.

## Key Signaling Pathways Altered in Neurodegenerative Disorders

### Wnt/Wg β-catenin signaling pathway

The Wnt/Wg β-catenin pathway is an evolutionarily conserved crucial pathway that regulates cell migration and fate (Logan and Nusse, 2004; De Gregorio et al., 2018; Ramakrishna et al., 2023). The Wnt/Wg β-catenin pathway is key for growth, early neuronal development, synaptic plasticity, and survival (Logan and Nusse, 2004; Libro et al., 2016; Sarkar et al., 2016). In this pathway, Wnt/Wg proteins bind to Frizzled receptors and stabilize β-catenin, which then translocates to the nucleus and activates genes involved in cell growth and survival (Logan and Nusse, 2004). In several model systems, including *Drosophila*, studies show that neurodegenerative disease pathogenesis triggers aberrant signaling (Logan and Nusse, 2004; Sarkar et al., 2016; Sarkar et al., 2018; Ramakrishna et al., 2023; Deshpande et al., 2024).

#### miR-9

As discussed in prior sections, miR-9 is profusely expressed in the developing brain. Further, miR-9 has several cell-autonomous effects on NPC migration, differentiation, and survival, and modulates various mRNAs involved in neurodegenerative disorders (Cai et al., 2009; Coolen et al., 2013; **Figure 2** and **Table 1**). As previously discussed miR-9 plays a key role in modulating AD, HD, and ALS (Packer et al., 2008; Chang et al., 2017; Di Pietro et al., 2017; Chen et al., 2021). In AD, miR-9 has been shown to regulate APP processing and Aβ accumulation, which is linked to the Wnt/Wg β-catenin pathway (Coolen et al., 2013; Wittkorn et al., 2015; Sarkar et al., 2016; Chen et al., 2021; Ramakrishna et al., 2023). miR-9 downregulation impairs this pathway, leading to synaptic dysfunction and neuronal death. miR-9 also regulates Clusterin, an AD-associated protein, that targets Dickkopf-1, a well-known Wnt/Wg β-catenin signaling inhibitor (Libro et al., 2016). The soluble amyloid-β promotes intracellular accumulation of Clusterin and subsequent upregulation of Dickkopf-1. Clusterin knockdown prevents Dickkopf-1 induction and is protective against Aβ neurotoxicity (Libro et al., 2016).

In HD, several Wnt/Wg signaling pathway components such as β-catenin are dysregulated including the APC regulator of the WNT signaling pathway (*APC*) and glycogen synthase kinase 3β (*GSK3*β) (Smith-Geater et al., 2020). Normally, Wnt/Wg β-catenin signaling promotes NPC differentiation; however, in ALS upstream effector molecules such as phosphorylated GSK–3β are upregulated and thus hyperactivate Wnt/Wg β-catenin signaling (Jiang et al., 2021). Mechanisms identifying how miR-9 directly impacts Wnt/Wg β-catenin signaling remain unclear.

#### miR-34

As previously discussed, miR-34 manages NSC/NPC differentiation and neuronal maturation (Agostini et al., 2011; Aranha et al., 2011; Choi et al., 2011; De Antonellis et al., 2011; Jauhari et al., 2018; **Figure 2** and **Table 1**). miR-34 directly activates Wnt/Wg β-catenin signaling by inhibiting Wnt binding to the Frizzled receptor and co-receptor LDL receptor-related protein, β-catenin stabilization by Axin, β-catenin nuclear entry, and/or β-catenin interaction with TCF for downstream gene activation (Peng et al., 2017). Given the multiple modulatory points, in neurodegenerative disorders, these microRNAs likely play a role in pathway signaling and should be further explored.

### Mitogen-activated protein kinase/extracellular signal-regulated kinase signaling pathway

The mitogen-activated protein kinase/extracellular signal-regulated kinase (MAPK/ERK) pathway is involved in regulating cell growth, survival, and differentiation (Dajas-Bailador et al., 2012; Banzhaf‐Strathmann et al., 2014). This pathway is crucial for synaptic plasticity, memory, and learning. However, aberrant activation of the MAPK/ERK pathway has been implicated in neurodegenerative disorders, where it contributes to neuroinflammation, tau hyperphosphorylation, and neuronal loss (Banzhaf‐Strathmann et al., 2014).

#### miR-21

In neurodegenerative disorders such as AD and PD, miR-21 is often upregulated and has been shown to activate the MAPK/ERK pathway (Mei et al., 2013; Bai and Bian, 2022; Garcia et al., 2022; **Figure 2** and **Table 1**). MAPK/ERK activation can exacerbate neurodegeneration via tau hyperphosphorylation and neuronal apoptosis (Dajas-Bailador et al., 2012; Mei et al., 2013; Banzhaf‐Strathmann et al., 2014; Garcia et al., 2022). Studies demonstrate that miR-21 upregulation can inhibit Aβ-induced apoptosis by enhancing phosphatidylinositol 3-kinase/protein kinase B pathway and GSK-3β protein levels (Bai and Bian, 2022; Garcia et al., 2022). Additionally, miR-21 also represses Sprouty2 (*SPRY2*) expression, which alters the receptor tyrosine kinase signaling pathway. Modulations in receptor tyrosine kinase activity heavily impact the progression of several Neurodegenerative disorders including AD and PD (Mei et al., 2013). Receptor tyrosine kinase modulation further impacts MAPK/ERK duration, and aids in cellular protection.

#### miR-125b

miR-125b downregulation negatively regulates MAPK/ERK signaling, and in AD miR-125b may promote MAPK/ERK overactivation, which contributes to neuronal dysfunction and inflammation (Banzhaf‐Strathmann et al., 2014; Zhang et al., 2017; Liu et al., 2024; **Figure 2** and **Table 1**). miR-125b upregulation directly correlates with increase in phosphorylated p44/42-MAPK and total p44/42-MAPK protein levels. These observations suggest an increase in kinase expression is likely linked to tau hyperphosphorylation due to miR-125b overexpression (Banzhaf‐Strathmann et al., 2014).

### Nuclear factor-κB signaling pathway

NF-κB signaling regulates immune and inflammatory responses and alterations in this pathway trigger the pathogenesis of several diseases including Neurodegenerative disorders (Choi et al., 2011; Saba et al., 2014; Lei et al., 2021). NF-κB signaling activates via canonical and noncanonical mechanisms. The canonical pathway responds to a diverse array of stimuli, while the noncanonical pathway is more selective (Liu et al., 2017). NF-κB pathway activation is proinflammatory and in the context of neurodegenerative disorders, it promotes neuroinflammation through various mechanisms, maintains a highly oxidative environment, and eventually chronic activation leads to neuronal death (Choi et al., 2011; Saba et al., 2014; Lei et al., 2021).

#### miR-146a

miR-146a is upregulated in AD and inhibits tumor necrosis factor receptor-associated factor 6 cytoplasmic protein that acts on other downstream protein complexes and influences NF-κB degradation (Saba et al., 2014). Upon miR-146a mediated NF-κB degradation, inflammation decreases. NF-κB signaling is often mediated by toll-like receptors that promote proinflammatory environments (Ammal Kaidery et al., 2021). Proinflammatory environments also allow for the formation of high levels of reactive oxygen species (Deshpande et al., 2021, 2023, 2024). Increases in reactive oxygen species are hallmarks of several Neurodegenerative disorders including AD, PD, and ALS (Choi et al., 2014; Anderson et al., 2018; Jauhari et al., 2018; Gogia et al., 2020; Irwin et al., 2020; Ammal Kaidery et al., 2021; Lei et al., 2021; Singh et al., 2024).

#### miR-155

miR-155 regulates microglial activation and has been found to be upregulated in AD and PD (Ammal Kaidery et al., 2021; Zingale et al., 2021). miR-155 is encoded by the B-cell integration cluster (*BIC*) gene and its activation leads to chronic inflammation that exacerbates neuronal injury. miR-155 targets the toll-like receptor associated with NF-κB signaling resulting in the release of proinflammatory cytokines such as interferon-gamma while simultaneously targeting anti-inflammatory regulators such as the suppressor of cytokine signaling (*SOCS1*) gene (Zingale et al., 2021). Together, miR-155 generates a highly inflamed environment and utilizes components of the NF-κB signaling pathway to progress neurodegenerative disorders.

## Therapeutic Potential of MicroRNAs in Neurodegenerative disorders

MiRNAs offer promising therapeutic potential for the treatment of neurodegenerative diseases, as these small molecules have the ability to regulate multiple genes involved in disease progression. The therapeutic potential of miRNAs, based on their ability to modulate complex molecular pathways, can be harnessed to slow down or reverse the progression of neurodegenerative disorders (Packer et al., 2008; Di Pietro et al., 2017; Sheinerman et al., 2017; Waller et al., 2017; Zhang et al., 2017; Joilin et al., 2019; Han et al., 2020).

### Role as biomarkers

MicroRNAs are placed in a unique position as they are readily observable in serum samples and have been shown to react to pathological changes seen in Neurodegenerative disorders (Waller et al., 2017; Joilin et al., 2019; Ramakrishna et al., 2023). miR-9 is a clear example of an abundant microRNA that displays changes in expression levels upon disease onset and can be used as a clear indicator for neurodegeneration (Packer et al., 2008; Coolen et al., 2013; Hawley et al., 2019; Chen et al., 2021). Specifically, miR-133b is consistently decreased in PD serums (Heyer et al., 2012; Zhang et al., 2017). miR-143 is observed in sALS patient serum samples and studies suggest that miR-143 is linked to increased muscle denervation of motor neurons during disease progression (Waller et al., 2017; Joilin et al., 2019). miR-125b is also consistently differentially expressed in AD patient serums compared to normal (Liu et al., 2024). Therefore, if specific Neurodegenerative disorders are associated with varied miRNA levels, early detection for Neurodegenerative disorders is a future possibility. Using miRNA as serum markers as a diagnostic tool would aid in ND management. miRNAs are detectable using known assays such as quantitative reverse transcription polymerase chain reaction, making them accessible with today’s technology.

### MicroRNAs as treatment options

MicroRNAs may be synthetically designed to mimic the function of known downregulated microRNAs to restore pathological regulation. Mimics such as amiRNAs, or artificial miRNAs, can endogenously act on other miRNAs or mRNA sequences in place of the downregulated miRNA. For instance, miR-133b mimics could be used to restore the function of the parkin gene in PD (Heyer et al., 2012; Zhang et al., 2017). miR-29 downregulation in AD increases Aβ levels (Liu et al., 2016); therefore, miR-29 mimics could potentially reduce Aβ present. In ALS, miR-206 suppression downregulates HDAC4, hastening motor neuron dysregulation (Williams et al., 2009). Here, utilizing a mimic to upregulate miR-206 levels could reduce motor neuron dysregulation. miR-143 downregulation promotes myoblast differentiation and contributes to muscle-wasting diseases such as ALS (Waller et al., 2017). Again, miR-143 mimics would help alleviate muscle wasting by reducing myoblast differentiation.

Interestingly, miR-106b downregulation reduces Aβ formation by ABCA1 repression (Liu et al., 2016). Here, a promotion of downregulation is beneficial. Therefore, a short hairpin miRNA or silencing miRNA could further reduce miR-106b levels or artificial mimics that also repress ABCA1 can be utilized to reduce Aβ formation.

Both silencing miRNAs and short hairpin miRNAs fall under a broader category of microRNAs called anti-miRs. Anti-miRs function to reduce or inhibit the generation of specific microRNAs that are overexpressed or associated with neurodegenerative disorders. Generating anti-miRs could aid in restoring homeostatic states such as inhibiting miR-34a in AD could reduce tau hyperphosphorylation and improve cognitive function (Aranha et al., 2011; Choi et al., 2011). Overexpression of miR-133b in PD reduces mature DN number, compromising the fidelity of communication between the striatum and basal ganglia (Zhang et al., 2017). Here, an anti-miR would help in reducing miR-133b levels and restoring communication between brain structures.

Further, in recent years, sponge miRNA, which contains complementary binding sites to a miRNA of interest, has evolved one more mode of therapeutic methods to inhibit miRNA function (Carè et al., 2007; Ebert et al., 2007). Sponge miRNA when expressed, specifically hampers the activity of a family of miRNAs, which has a common seed sequence, resulting in the blocking of a whole family of related miRNAs. A study from miRNA sponge has revealed that miR-330 sponge negatively regulates the NF-kB signaling pathway in PD, by targeting SHIP1, inhibiting M1 microglia polarization, and improving motor dysfunction by repairing dopaminergic neurons (Feng et al., 2021). The efficiency of the miRNA sponge depends on both the affinity of binding sites, and on the high amount of sponge RNAs relative to the amount of the miRNA, which can be achieved by expressing the sponge from strong promoters such as the CMV promoter.

Given the broad capacity for preventing further disease pathology, using miRNA mimics or anti-miRs may be a novel frontier for pharmaceutical research. These innovations could pave the way for more personalized and effective treatment regimens for neurodegenerative disorders. There are still challenges in improving delivery methods and ensuring the effectiveness of these treatment regimens. However, advances in the field of medicine could lead to more effective miRNA-based personalized therapies in the future.

## Conclusion

MiRNAs are a new frontier for understanding how post-translational modulation can play a role in systemic neurodegenerative pathology. The ability of microRNAs not only to regulate the 3’UTR of proteins, but also other miRNAs including themselves makes them a highly capable regulator (Packer et al., 2008). MiRNAs function in larger signaling pathways that are often impaired in neurodegenerative diseases (Mei et al., 2013; Libro et al., 2016; Liu et al., 2017; Peng et al., 2017; Deshpande et al., 2024). Dysregulated miRNA expression can contribute to the pathogenesis of neurodegenerative diseases by affecting processes such as protein homeostasis, inflammation, synaptic function, and cellular survival. Understanding the complex interplay between miRNAs and signaling pathways is crucial for developing effective therapeutic interventions that can address the underlying molecular causes of neurodegenerative disorders. Therefore, using miRNAs as biomarkers and utilizing their modulatory ability to generate synthetic treatment options is where pioneering pharmaceutical technologies should focus their efforts. Here, miRNAs can potentially correct aberrant signaling and slow down or halt the progression of neurodegenerative disorders. As such, understanding the role of miRNA in disease and therapeutic capacity continues to be an area of great interest in the scientific community. Thus, with the latest advances in precision medicine and RNA-based therapies, miRNA research offers promising opportunities for diagnostics and treatment of neurodegenerative disorders and other diseases. This review highlights some of the great scientific contributions made in understanding miRNAs and their role in neurodegenerative pathology and progression.

## Data Availability

*Not applicable*.
